# Fabrication of 50.0 μm Ultra-Fine Pure Rhodium Wire, Using a Multi-Pass Wire Drawing Process, for Probe Card Pins

**DOI:** 10.3390/ma12132194

**Published:** 2019-07-08

**Authors:** Sang-Kon Lee, In-Kyu Lee, Sung-Yun Lee, Sun-Kwang Hwang

**Affiliations:** Extreme Fabrication Technology Group, Korea Institute of Industrial Technology, 320 Techno sunhwan-ro, Yuga-up, Dalseong-gun, Daegu 42994, Korea

**Keywords:** multi-pass wire drawing process, process design, ultra-fine pure rhodium wire, probe card pin

## Abstract

Rhodium is a rare material that is widely used in electrical and electronic components due to its excellent mechanical and electrical properties. Ultra-fine rhodium wires in particular are widely used in electronic components. In this study, a multi-pass wire drawing process was designed to fabricate ultra-fine pure rhodium wire with a diameter of 50.0 µm from an initial diameter of 80.0 µm, which is used as probe card pins. An elastic–plastic finite element (FE) analysis was performed to validate the pass schedule that was designed for this study. A fine wire drawing experiment was also carried out to verify the effectiveness of the designed process. As a result, the ultra-fine rhodium wire was fabricated using the design process without wire breaks and the diameter of the final drawn wire was 47.80 µm.

## 1. Introduction

**Figure 1:**
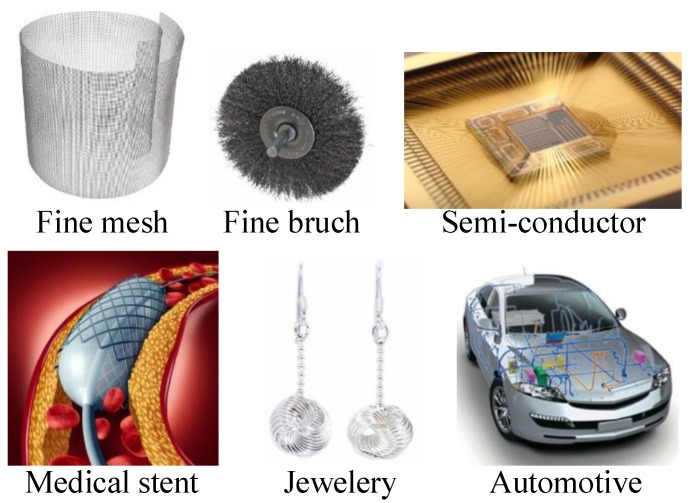
Representative applications of very fine wire.

Fine wires are manufactured from several different materials, such as tungsten, copper, stainless steel, magnesium, and gold by a multi-pass wire drawing process [1,2,3,4,5,6,7,8]. Fine wires have a wide array of applications in many industries, as shown in Figure 1. Recently, ultra-fine wires are also being used in high value-added industries, such as electronics and medical devices [9,10,11].

Rhodium is a very hard rare material with a tensile strength of over 2000 MPa and an elastic modulus of about 360 GPa [12]. In the past, rhodium was mainly used in jewelry making, but it is now also being used in high-tech industries, such as in automotive catalysts and electronic components. The demand for fine rhodium wire has increased, as it is a pin material used to measure the electric characteristics of semiconductors or printed circuit boards (PCBs).

**Figure 2:**
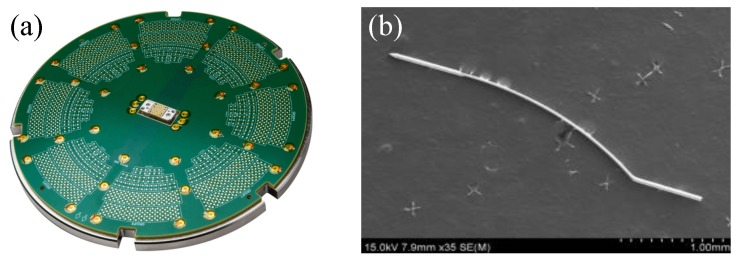
Photos of (**a**) probe card and (**b**) card pin.

A probe card is the interface module between an electronic test system and a semiconductor wafer that measures the micro-current of semiconductors [13]. As shown in Figure 2, there are many pins in a probe card that detect defects by measuring the micro-current of a semiconductor. It is important that the durability and the elasticity of the pin material should be excellent to achieve the test cycle and measure the micro-current through sufficient contact, respectively. Also, in order to measure the micro-current, a sufficient electrical conductivity is required. Until recently, tungsten, Ag–Pd alloys, and Be–Cu alloys have been used as pin materials. However, tungsten has relatively poor formability and electrical conductivity. Although Ag–Pd and Be–Cu alloys have excellent electrical conductivity, they have poor elasticity and cannot support a repeated load, making rhodium one of the best materials to overcome these problems.

The objective of this study is to fabricate 50.0 µm ultra-fine pure (99.9%) rhodium wire from the initial 80.0 µm rhodium wire using a multi-pass wire drawing process. Some studies have been carried out to design a multi-pass wire drawing process. Lee et al. designed the taper down reduction ratio pass to improve the drawing speed for high carbon steel wire [14]. Rodriguez-Alabanda et al. developed an expert system for the optimization of the multi-stage copper wire drawing process to reduce energy consumption [15]. In the wire drawing process for ultra-fine rhodium, the temperature rise is not a problem because the drawing speed is very low. In addition, lubricants are not applied because they cause wire breaks during the subsequent heat treatment process. Moreover, in the ultra-fine wire drawing process, the drawing load is very low. Therefore, the energy consumption is not an important issue. Therefore, in this study, the uniform reduction ratio theory was applied to design the multi-pass wire drawing process to fabricate the 50.0 µm ultra-fine pure rhodium wire. Then, elastic–plastic finite element (FE) analysis was carried out to validate the designed pass schedule [16]. In FE analysis, the deformation history of the previous pass was considered to analyze the next pass for improving the accuracy of analysis [17]. Through FE analysis, the wire diameters and drawing stresses were evaluated at each pass. Further, a precision wire drawing machine was developed specifically for this experiment. Finally, an ultra-fine wire drawing experiment was performed using the developed precision wire drawing machine.

## 2. Materials and Methods

### 2.1. Pass Schedule of the Multi-Pass Wire Drawing Process

It is very important to design an appropriate pass schedule in the multi-pass drawing process for fabricating fine wires, as the application of an inappropriate pass schedule could lead to wire breakage, and resuming the process is fairly expensive and time consuming [14].

Two pass schedule methods can be used depending on the set of the reduction ratio of each pass [18]. The first method is the uniform-reduction ratio method, wherein the reduction ratio of all passes is constant. The other method is a taped-reduction ratio method, with different reduction ratios for each pass. Although this method is applied to control the wire temperature or the drawing power at each pass, it is more complicated than the uniform-reduction ratio method [18]. In an ultra-fine wire drawing process, the wire temperature does not rise excessively due to low drawing speed. Therefore, in this study, the uniform-reduction ratio method was applied as follows:

(1) $$r_{avg}=\left[1-{\left(1-\frac{r_{t}}{100}\right)}^{1/n}\right]\times 100\;\left(\%\right)$$

### 2.2. FE Analysis Conditions and Material Properties

**Figure 3:**
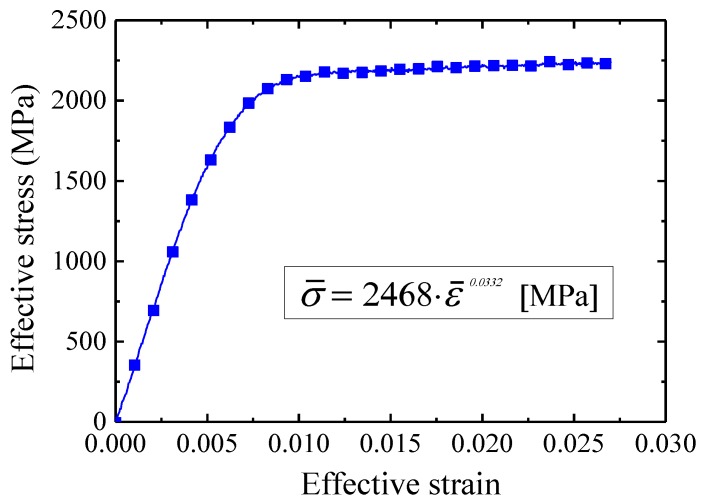
Flow stress curve of the initial rhodium wire.

An elastic–plastic FE analysis was carried out to validate the designed uniform-reduction ratio pass schedule. Firstly, a tensile test was performed to obtain the flow stress curve of the initial 80.0 µm wire and the mechanical properties of the initial wire. A low capacity universal tensile test system (Instron 5566A) was applied for the tensile test. Figure 3 and Table 1 show the obtained flow stress curve and other mechanical properties of the initial rhodium wire.

**Figure 4:**
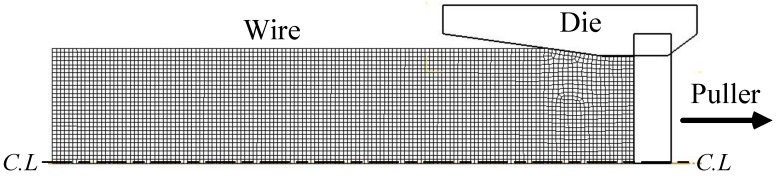
Initial model of the elastic–plastic analysis.

Figure 4 is the initial FE analysis model. DEFORM-2D software (Ver. 11.2, SFTC, Columbus, USA) was used for the analysis. In the analysis, the wire is an elastic–plastic material and the other tools are rigid bodies [19].

### 2.3. Friction Coefficient

In the wire drawing process, the *μ* between the wire and die is one of important process factors. The *μ* should be obtained for improving the accuracy of analysis. In this study, the *μ* was obtain using the drawing load prediction model as follows [20]:

(2) $$L=k_{m}\left(F+Q\cdot \mu \right)+0.77\cdot k_{fm}\cdot f_{2}\cdot \alpha .$$

From Equation (2), the *μ* can be calculated through the following equation:

(3) $$\mu =\frac{1}{Q}\left[\frac{\left(L-0.77\cdot k_{fm}\cdot f_{2}\cdot \alpha \right)}{k_{m}}-F\right].$$

**Figure 5:**
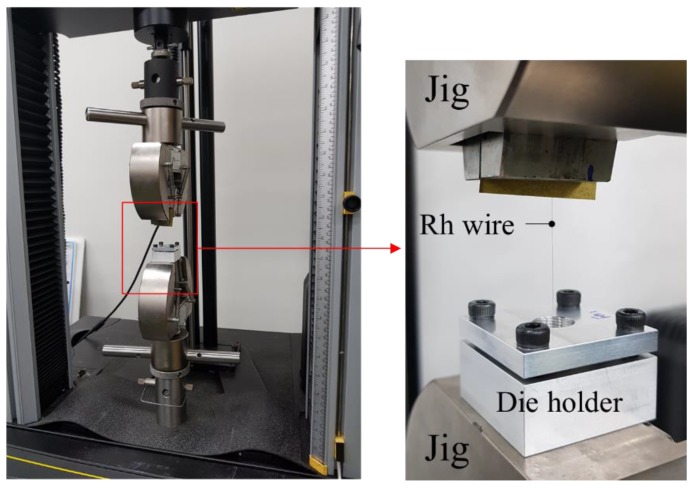
Equipment (Instron 5566A) for measuring the drawing load.

In this study, a non-lubrication condition was applied. In order to obtain the *μ*, a single pass drawing experiment was performed to measure the *L*. Figure 5 shows the experimental equipment (Instron 5566A) for measuring the *L*. The designed first pass was applied to measure the *μ*. Table 2 shows the conditions of the single pass drawing experiment. The *L* was 4.1 N. Therefore, the calculated *μ* was 0.0589. This value was applied to the FE analysis.

### 2.4. Drawing Die and the Precision Fine Wire Drawing Machine

In order to validate the design wire drawing process, a drawing experiment was carried out. Drawing dies and a precision single pass wire drawing machine were manufactured for the experiment.

**Figure 6:**
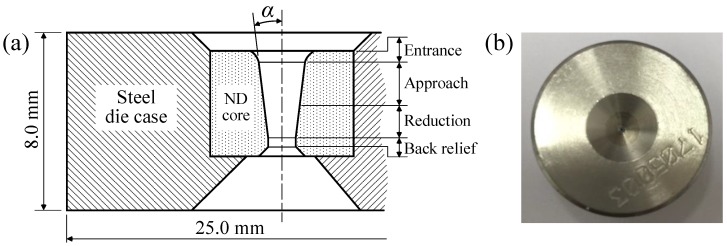
Wire drawing dies. (**a**) structure; (**b**) manufactured drawing die. ND, natural diamond.

Natural diamond (ND) dies were manufactured, as shown in Figure 6. The ND core for the deformation of wire is embedded in the steel die case.

**Figure 7:**
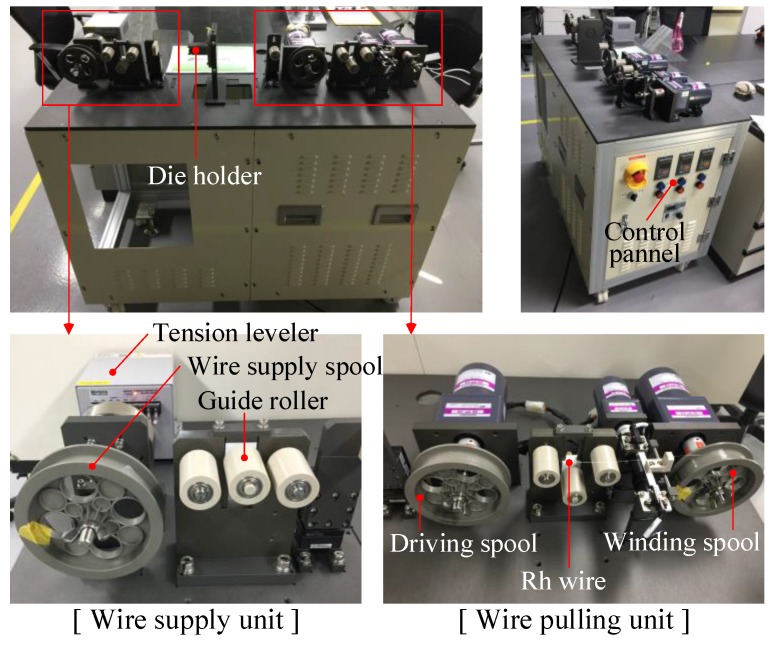
Precision fine wire drawing machine.

A precision fine wire drawing machine was also designed and manufactured for the fine wire drawing experiment. Figure 7 shows the manufactured precision drawing machine. There is a tension leveler at the wire supply unit to control the tension of the supplied wire. The wire passes through the drawing die by the pulling force of the driving spool. Finally, the drawn wire is wound around the winding spool.

## 3. Results and Discussion

### 3.1. Designed Pass Schedule

The initial diameter of the rhodium wire is 80.0 µm. Therefore, the *r_t_* is 60.94%. The *n* was set to 8 based on the know-how of real industries. Therefore, the *r_avg_* was about 11.1%. Table 3 shows the wire diameter at each pass. The semi-die angle is 8.0°, and the bearing length is 0.3 times of the inlet wire diameter

### 3.2. Result of the FE Analysis

**Figure 8:**
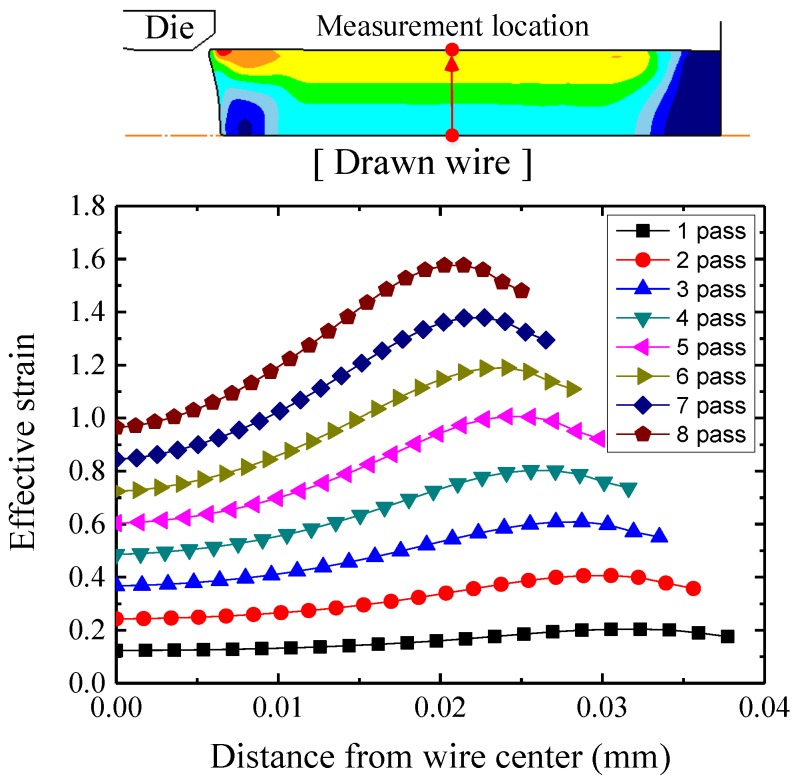
Distribution of the effective stain in the drawn wire.

Figure 8 shows the distribution of effective strains at each pass. The strain increases gradually according to the pass number as the wire diameter decreases. The minimum strain is in the center of the wire. The strain of the region near the surface is higher than that near the center because the surface is in direct contact with the drawing die. Also, the strain difference between the center and near surface regions increases according to the pass number because of the increase in non-uniform deformation. Therefore, in the first and the last pass, the maximum differences of strain between the center and near surface were 0.0799 and 0.6111, respectively.

**Figure 9:**
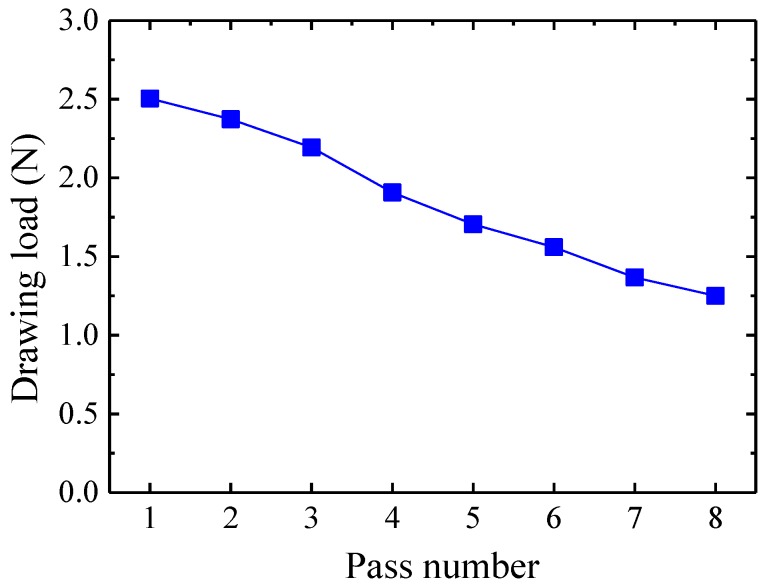
Drawing load.

Figure 9 shows the drawing load of the FE analysis. Although the strain hardening increases according to the pass number, the drawing load decreases according to the decrease in wire diameter.

In the wire drawing process, if the stress is greater than the yield strength of the material at the die exit, excessive plastic deformation or wire breaks occur. Therefore, in this study, the average drawing stress at the die exit was calculated to assess the occurrence of plastic deformation. Based on the drawing load and the cross-sectional area of the die exit, the average drawing stress can be calculated by

(4) σD,i=LiAi

where *i* is the pass number.

**Figure 10:**
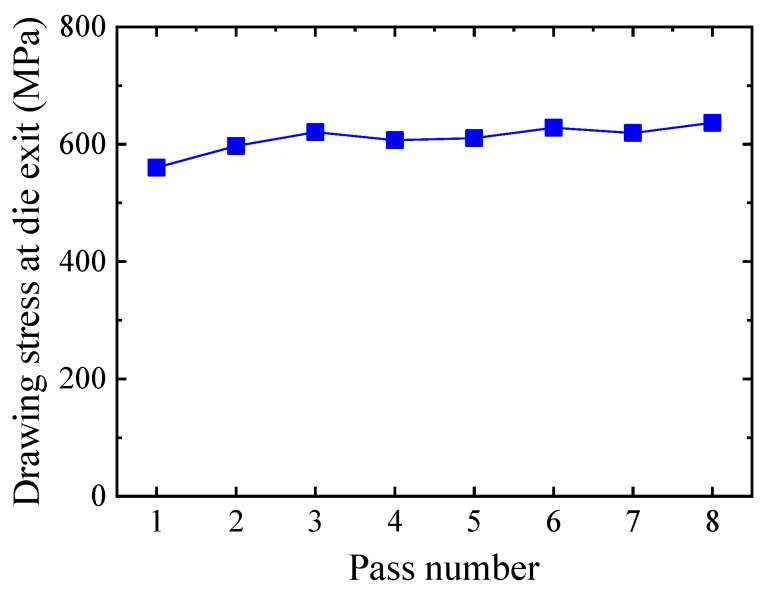
Drawing stress at the die exit.

Figure 10 shows the calculated average drawing stress at the die exit. As shown in Figure 10, the drawing stresses are not different between 560 and 636 MPa because the cross-sectional area of the drawn wire decreases according to the pass number. All the drawing stresses are below the yield strength of the initial wire. Therefore, it can be seen that excessive deformation does not occur at each pass after drawing.

### 3.3. Result of Wire Drawing Experiment

**Figure 11:**
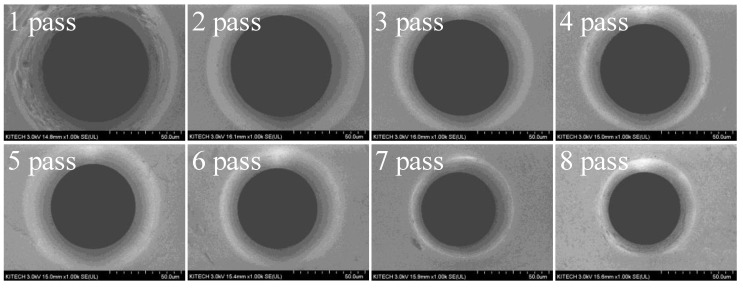
Wire drawing dies.

Figure 11 shows the SEM (SU8020, Hitachi, Japan) image of the drawing dies. In order for the pointed wire to pass through the drawing die well, the surface condition of the approach area of die should be excellent. As shown in Figure 11, the surface of the first die is relatively worse than that of the other dies.

**Figure 12:**
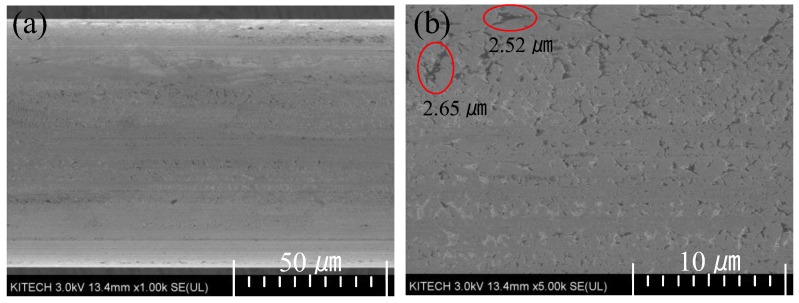
SEM image of the initial wire. (**a**) ×1000; (**b**) ×5000.

Figure 12 shows an SEM image of the initial 80.0 µm pure rhodium wire. As shown in Figure 12, there are many micro-cracks of mostly 2.65 µm on the surface of the initial wire. In the multi-pass wire drawing process, a micro-crack is one of the main causes of wire breakage because the breakage can begin or progress at the micro-crack.

**Figure 13:**
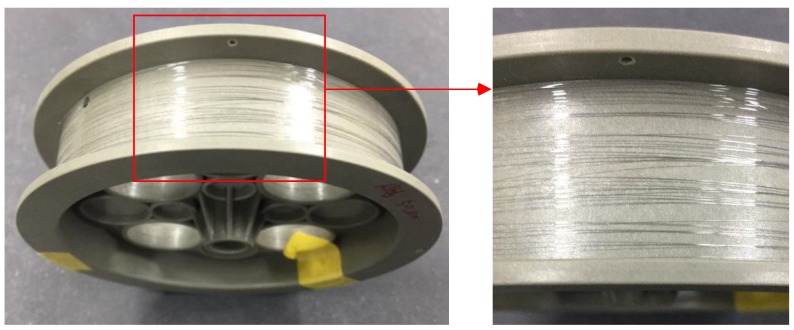
Final drawn wire.

Figure 13 is the final drawn wire. Throughout the experiment, the final drawn wire could be produced without wire breakage.

**Figure 14:**
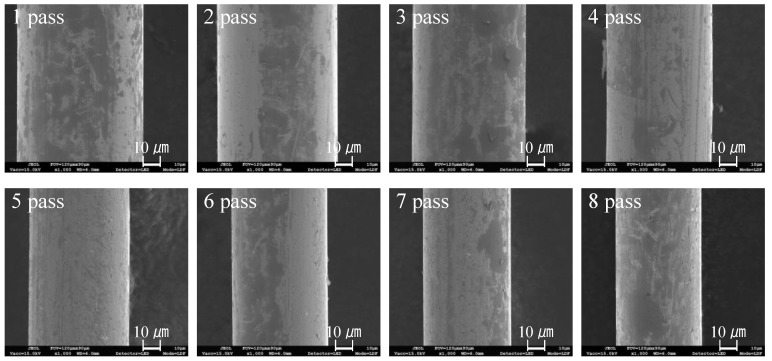
SEM image (×1000) of the drawn wire at each pass.

A SEM image of the drawn wire at each pass is shown in Figure 14. Although micro-cracks were observed at all passes, no wire breakage was present. In the wire drawing process, the tensile deformation mode is mainly applied to the wire. Therefore, large micro-cracks cause wire breakage. Thus, it is known that initial micro-cracks less than 1.35 µm in size did not cause wire breakage. Further research is required to determine the critical initial micro-crack size that causes wire breakage.

After the wire passes through the drawing dies, if excessive plastic deformation or elastic recovery occurs, the diameter of the drawn wire changes extensively. This is a very serious problem. Therefore, the diameter of the drawn wire was evaluated. Table 4 shows the comparison of the diameter of the drawn wire between the FE analysis and the experiment. As shown in Table 4, there is no excessive plastic deformation or elastic recovery after drawing. Further, it can be seen that the results are in good agreement with each other.

**Figure 15:**
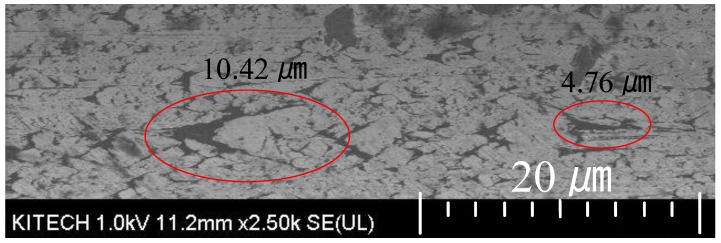
SEM image (×2500) of the final drawn wire.

Figure 15 shows the surface of the final drawn wire. As shown in Figure 15, there are micro-cracks on the surface. During the drawing process, the maximum crack size increased from 2.65 µm to 10.42 µm due to the tensile deformation mode of wire and the friction between the wire surface and the die. Although the crack size is somewhat large in the axial direction, it is believed that wire breakage did not occur because of the narrow width and shallow depth of the cracks. In the multi-pass wire drawing process, the crack width decreased because of the circumferential compression in the drawing die. In addition, the crack size increased in the axial direction, and the crack depth gradually deceased [21,22]. This means that if the crack size and depth of the initial wire are less than the critical value, the crack can be eliminated. Therefore, the initial surface condition of the wire is very important for preventing wire breakage in the multi-pass wire drawing.

**Figure 16:**
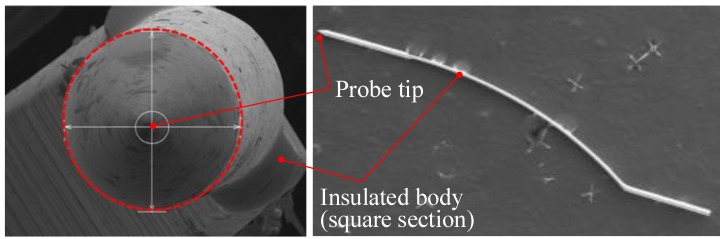
Shape of the probe card pin after press forming.

After the wire drawing process, a press forming was applied to fabricate the probe card pin. As shown in Figure 16, the bended square insulated body was formed through the press forming press. If the diameter exceeds 50.0 µm, press forming is impossible, as the wire position is incorrect in the forming die. The diameter of the final wire, measured using SEM, was 47.80 µm.

## 4. Conclusions

As the demand for fine parts has gradually increased over recent years, the demand for ultra-fine wires (which are important components of electric or electronic products, including semi-conductors) has also increased. In this study, a 50 µm ultra-fine pure rhodium wire, which is used for probe card pins, was fabricated by a multi-pass wire process. The following conclusions were obtained:

(1) Based on the uniform reduction ratio theory, a multi-pass wire drawing process was designed to fabricate a 50 µm rhodium wire from an 80 µm initial wire. The total pass number was set at 8. As a result, the average reduction ratio of each pass was 11.1%.

(2) In order to evaluate the friction coefficient between rhodium wire and the drawing die, a single pass drawing experiment was carried out. Based on the experimental result and the drawing load prediction model, the calculated friction coefficient was 0.0589. This value was applied to perform the FE analysis.

(3) From the elastic–plastic FE analysis, the drawing stress of each pass was between 560 MPa and 636 MPa. Because the drawing stress was less than the yield strength of the initial wire, there would be no plastic deformation in the wire if the wire passes through the drawing dies.

(4) From the wire drawing experiment, a 50 µm ultra-fine rhodium wire could be fabricated using the designed pass schedule without wire breakage. The final wire diameter was 47.80 µm. In the final drawn wire, the maximum surface crack increased from 2.65 µm to 10.42 µm in the axial direction. Although the size increased by approximately four times, there was no wire breakage at any pass.

## Figures and Tables

**Table 1:** Mechanical properties of the initial rhodium wire.

Item	Value
Young’s modulus (GPa)	359.0
Poisson’s ratio	0.26
Yield strength (MPa)	1790.0
Tensile strength (MPa)	2190.0

**Table 2:** One pass drawing experiment conditions for measuring the friction coefficient (*μ*).

Item	Value
Inlet wire diameter (mm)	0.085
Exit wire diameter (mm)	0.075
Semi-die angle (^o^)	8.0
*f_2_* (mm^2^)	0.00442
*F* (mm^2^)	0.00126
*k_fm_* (MPa)	2178.3
*k_m_* (MPa)	1714.3

**Table 3:** The designed pass schedule.

Pass No.	Diameter of Wire (μm)	Reduction Ratio (%)	Semi-Die Angle (^o^)	Bearing Length
1	75.0	11.1	8.0	0.3 D_i_ (D_i_: inlet wire diameter)
2	71.0			
3	67.0			
4	63.0			
5	60.0			
6	56.0			
7	53.0			
8	50.0			

**Table 4:** Comparison of the wire diameter at each pass.

Pass No.	Diameter of FEA (μm)	Diameter of Experiment (μm)
1	75.000	74.675
2	70.970	71.020
3	66.980	67.208
4	63.180	62.987
5	59.850	60.065
6	56.200	56.169
7	53.010	52.922
8	48.010	47.800

## Nomenclature

$\bar{\sigma }$	effective stress
$\bar{\varepsilon }$	effective strain
*r_ave_*	average reduction ratio
*r_t_*	total reduction ratio
*n*	total number of passes
$\sigma_{D}$	Drawing stress
*L*	Drawing load
*A*	cross sectional area of die exit
*μ*	friction coefficient
*α*	Semi-die angle
*k_fm_*	mean yield strength of before and after drawn wire
*k_m_*	average deformation resistance of wire
*F*	difference in the cross-sectional area of wire at the inlet and the exit of die
*f_2_*	cross-sectional area of the die exit
*Q*	Contact area between wire and die
